# Covert spatial attention is uniform across cardinal meridians despite differential adaptation

**DOI:** 10.1167/jov.26.1.15

**Published:** 2026-01-28

**Authors:** Hsing-Hao Lee, Marisa Carrasco

**Affiliations:** 1 Department of Psychology, New York University, New York, NY, USA; 2 Center for Neural Sciences, New York University, New York, NY, USA

**Keywords:** visual adaptation, endogenous attention, exogenous attention, visual performance asymmetries, contrast sensitivity

## Abstract

Visual adaptation and attention are two processes that help manage the limited bioenergetic resources of the brain for perception. Visual perception is heterogeneous around the visual field: It is better along the horizontal than the vertical meridian (horizontal–vertical anisotropy [HVA]), and better along the lower than the upper vertical meridian (vertical meridian asymmetry [VMA]). Recently, we showed that visual adaptation is more pronounced at the horizontal than the vertical meridian, but whether and how this differential adaptation modulates the effects of covert spatial attention remain unknown. In this study, we investigated whether and how the effects of endogenous (voluntary) and exogenous (involuntary) covert attention on an orientation discrimination task vary at the cardinal meridians, with and without adaptation. We manipulated endogenous (Experiment 1) or exogenous (Experiment 2) attention via an informative central or uninformative peripheral cue, respectively. Results showed that (a) in the non-adapted condition, the typical HVA and VMA emerged in contrast thresholds; (b) the adaptation effect was stronger at the horizontal than the vertical meridian; and (c) regardless of adaptation, both endogenous and exogenous attention enhanced and impaired performance at the attended and unattended locations, respectively, to a similar degree at both cardinal meridians. Together, these findings reveal that, despite differences between endogenous and exogenous attention, their effects remain uniform across cardinal meridians—even under differential adaptation that reduces intrinsic asymmetries of visual field representations.

## Introduction

Visual adaptation and attention are two processes that optimize performance and help manage the limited bioenergetic resources of the brain by allocating them according to task demands (Carrasco, 2011; Lee, Fernández, & Carrasco, 2024; Lennie, 2003; Pestilli, Viera, & Carrasco, 2007). Although both processes modulate sensory responses, they have opposite effects on the contrast response function. Visual adaptation helps manage bioenergetic resources by increasing metabolic efficiency—it reduces sensitivity to repeated features and enhances sensitivity to novel ones. For example, contrast adaptation can adjust the gain of the neural response so that its dynamic range is matched to the range of levels in the stimulus (Boynton & Finney, 2003; Gardner et al., 2005; Kohn, 2007; Perini, Cattaneo, Carrasco, & Schwarzbach, 2012; Vergeer, Mesik, Baek, Wilmerding, & Engel, 2018; Webster, 2011; Webster, 2015). In contrast, visual attention selectively improves information processing at an attended location while impairing processing elsewhere—a ubiquitous performance tradeoff considered a push–pull mechanism (e.g., Dosher & Lu, 2000a; Ling & Carrasco, 2006a; Pestilli & Carrasco, 2005; Pestilli, Ling, & Carrasco, 2009; Pestilli et al., 2007; for reviews, see Carrasco, 2011; Carrasco, 2014; Desimone & Duncan, 1995; Olivers, 2025).

There are two types of covert spatial attention: endogenous and exogenous. Endogenous attention is voluntary, goal driven, and flexible; exogenous attention is involuntary, stimulus driven, and automatic. Endogenous attention requires ∼300 ms to be deployed and can be sustained for many seconds, whereas exogenous attention peaks at ∼120 ms and is transient (for reviews, see Carrasco, 2011; Carrasco, 2014). Despite these differences, both types of attention improve performance in many visual tasks, such as contrast sensitivity (e.g., Herrmann, Montaser-Kouhsari, Carrasco, & Heeger, 2010; Pestilli et al., 2009), appearance (for a review, see Carrasco & Barbot, 2019), and orientation discrimination (e.g., Fernández, Okun, & Carrasco, 2022). However, they have distinct effects in other tasks, such as texture segregation (e.g., Barbot & Carrasco, 2017; Jigo, Heeger, & Carrasco, 2021; Yeshurun & Carrasco, 1998), and they alter sensitivity across a different spatial frequency range (Fernández et al., 2022; Jigo & Carrasco, 2020).

**Figure 1.**
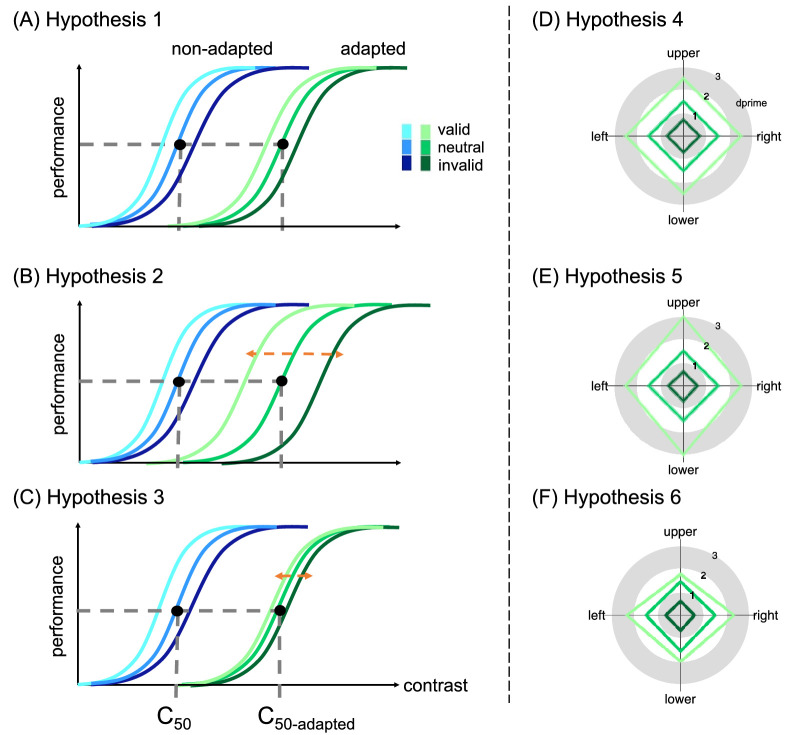
(**A**–**C**) Hypotheses regarding effects on contrast sensitivity. (**A**) Hypothesis 1: Attentional effect is comparable with and without adaptation. The C_50_ and C_50_-adapted values indicate the contrast threshold derived from the titration procedures in the non-adapted and adapted conditions, respectively. (**B**) Hypothesis 2: Attentional effect is larger with than without adaptation. (**C**) Hypothesis 3: Attentional effect is smaller than without adaptation. (**D**–**F**) Hypotheses regarding effects on contrast sensitivity as a function of location at the cardinal locations. Hypothesis 4: Attentional effect is comparable around polar angle after adaptation (**D**). Hypothesis 5: Attentional effect is stronger at the vertical than horizontal meridian (**E**). Hypothesis 6: Attentional effect is smaller at the vertical than horizontal meridian (**F**).

Exogenous attention restores contrast sensitivity after adaptation; although adaptation reduces sensitivity, the magnitude of the exogenous attentional benefit at the attended location and its concurrent cost at the unattended location remain comparable to those observed without adaptation (Lee et al., 2024; Pestilli et al., 2007). However, whether and how endogenous attention operates after adaptation are unknown. Thus, our first goal was to examine whether endogenous attention restores contrast sensitivity after adaptation. The following are possible after adaptation: (a) Endogenous attention enhances contrast sensitivity to a similar extent as without adaptation, assuming that, similar to exogenous attention (Lee et al., 2024; Pestilli et al., 2007), endogenous attention and adaptation yield independent effects on contrast sensitivity (Hypothesis 1) (Figure 1A). (b) Endogenous attention enhances sensitivity more than before adaptation, reflecting a compensatory process given the flexible nature of endogenous attention, which optimizes performance as a function of task demands (Barbot & Carrasco, 2017; Barbot, Landy, & Carrasco, 2012; Giordano, McElree, & Carrasco, 2009; Hein, Rolke, & Ulrich, 2006; Yeshurun, Montagna, & Carrasco, 2008), and it may help more than without adaptation, as after decreases there is more room for improvement (Hypothesis 2) (Figure 1B). (c) Endogenous attention enhances sensitivity less than without adaptation, if reduced baseline sensitivity limits the push–pull effects of endogenous attention (Hypothesis 3) (Figure 1C).

Because endogenous attention is flexible (Barbot & Carrasco, 2017; Barbot et al., 2012; Giordano et al., 2009; Hein et al., 2006; Yeshurun et al., 2008), but exogenous attention is not (Barbot et al., 2012; Carrasco, Loula, & Ho, 2006; Crotty, Massa, Tellez, White, & Grubb, 2025; Giordano et al., 2009; Hein et al., 2006; Luck & Thomas, 1999; Yantis & Jonides, 1996; Yeshurun & Carrasco, 1998), and because distinct brain regions are critical for their effect—right frontal eye fields for endogenous attention (Fernández, Hanning, & Carrasco, 2023) and early visual cortex for exogenous attention (Fernández & Carrasco, 2020; Lee et al., 2024), where it interacts with adaptation (Lee et al., 2024)—it is possible that they exert different effects on contrast sensitivity after adaptation. Therefore, our second goal was to determine whether endogenous attention and exogenous attention have similar or different effects on contrast sensitivity following adaptation.

Finally, we investigated whether target location matters. In adult humans, visual performance is better at the horizontal than the vertical meridian (horizontal–vertical anisotropy [HVA]) and better at the lower than the upper vertical meridian (vertical meridian asymmetry [VMA]). These visual field asymmetries, known as performance fields, are present in many fundamental visual tasks, including contrast sensitivity (Abrams, Nizam, & Carrasco, 2012; Baldwin, Meese, & Baker, 2012; Cameron, Tai, & Carrasco, 2002; Carrasco, Talgar, & Cameron, 2001; Corbett & Carrasco, 2011; Fuller, Rodriguez, & Carrasco, 2008; Himmelberg, Winawer, & Carrasco, 2020; Lee & Carrasco, 2025; Purokayastha, Roberts, & Carrasco, 2021), visual acuity (Kwak, Hanning, & Carrasco, 2023; Montaser-Kouhsari & Carrasco, 2009), spatial resolution (Altpeter, Mackeben, & Trauzettel-Klosinski, 2000; Carrasco, Williams, & Yeshurun, 2002; Greenwood, Szinte, Sayim, & Cavanagh, 2017; Talgar & Carrasco, 2002), and motion (Fuller & Carrasco, 2009; Tünçok, Kiorpes, & Carrasco, 2025), as well as mid-level visual processes such as texture segregation (Barbot, Xue, & Carrasco, 2021; Greenwood et al., 2017; Talgar & Carrasco, 2002; Wang, Murai, & Whitney, 2020) and crowding (Greenwood et al., 2017; Kurzawski, Burchell, Thapa, Winawer, Majaj, & Pelli, 2023; Petrov & Meleshkevich, 2011), and high-level tasks, such as numerosity perception (Chakravarthi, Papadaki, & Krajnik, 2022), face perception (Afraz, Pashkam, & Cavanagh, 2010; Kim & Chong, 2024), word identification (Tsai, Liao, Hou, Jang, & Chen, 2024), and visual short-term memory (Montaser-Kouhsari & Carrasco, 2009).

These visual field asymmetries are resistant to endogenous attention (Purokayastha et al., 2021; Tünçok, Carrasco, & Winawer, 2025) and exogenous attention (Cameron et al., 2002; Carrasco et al., 2001; Roberts, Ashinoff, Castellanos, & Carrasco, 2018; Roberts, Cymerman, Smith, Kiorpes, & Carrasco, 2016), as well as to temporal attention (Fernández, Denison, & Carrasco, 2019). Thus, performance fields are not easily reshaped. On the contrary, presaccadic attention, which enhances the processing at the location of the impending saccade target, exacerbates performance asymmetries at the cardinal locations by enhancing contrast sensitivity the most at the horizontal meridian and the least at the upper vertical meridian (Hanning, Himmelberg, & Carrasco, 2022; Hanning, Himmelberg, & Carrusco, 2024; Kwak, Hanning, & Carrasco, 2025; Kwak, Zhao, Lu, Hanning, & Carrasco, 2024).

A recent study showed that visual adaptation is stronger at the horizontal than the vertical meridian, leading to more homogeneous perception by mitigating the HVA (Lee & Carrasco, 2025). It remains unknown, however, whether and how endogenous and exogenous attention reshape performance fields after such differential adaptation. Thus, our third goal was to investigate whether, following adaptation, covert spatial attention enhances contrast sensitivity (a) to the same extent at the cardinal meridians around polar angle, similar to without adaptation (Hypothesis 4) (e.g., Carrasco et al., 2001; Purokayastha et al., 2021; Roberts et al., 2016; Roberts et al., 2018; Tünçok et al., 2025) (Figure 1D); (b) more at the vertical than the horizontal meridian and more at the upper than the lower vertical meridian, acting as a compensatory mechanism to reduces asymmetries (Hypothesis 5) (Figure 1E); or (c) more where baseline performance is already better (i.e., the horizontal meridian) than where it is worse (i.e., vertical meridian, especially the upper vertical meridian), thereby exaggerating asymmetries (Hypothesis 6) (Figure 1F).

Both adaptation (Altan, Morgan, Dakin, & Schwarzkopf, 2025; Dao, Lu, & Dosher, 2006; Gardner et al., 2005; Perini et al., 2012; Pestilli et al., 2007) and endogenous attention (Dosher & Lu, 2000b; Ling & Carrasco, 2006a; Lu, Lesmes, & Dosher, 2002; Pestilli et al., 2009) primarily affect the contrast gain of the contrast response function (i.e., a shift in threshold) (Figure 1A), whereas exogenous attention primarily affects response gain (i.e., a shift in asymptote) (Fernández & Carrasco, 2020; Pestilli et al., 2009). Additionally, according to a prominent normalization model of attention (Reynolds & Heeger, 2009), exogenous attention can also affect contrast gain when the attentional window is wider than the stimulus size, and endogenous attention can also affect response gain when the attentional window is narrower than the stimulus size (Herrmann et al., 2010). In this study, to directly compare the two types of attention before and after adaptation at the cardinal meridians, we induced a larger attentional window in the exogenous attention experiment, enabling contrast gain effects predicted by the normalization model of attention by Reynolds and Heeger (2009).

In summary, we asked (a) whether and how endogenous attention restores contrast sensitivity following adaptation, (b) whether endogenous attention and exogenous attention have similar or distinct effects on contrast sensitivity before and after adaptation, and (c) whether these effects uniformly or differentially across the cardinal meridians around the visual field. These findings are essential for elucidating how the visual system engages adaptation and attention—two fundamental visual processes that manage limited bioenergetic resources—to optimize performance across locations that differ in intrinsic discriminability and in their corresponding representation in cortical surface area.

## Experiment 1: Endogenous attention

### Methods

#### Participants

Twelve adults (five females; age range, 24–36 years old), including author H-HL, participated in the experiment. All of them had normal or corrected-to-normal vision. Sample size was based on previous studies on adaptation (Lee et al., 2024), with an effect size of *d* = 1.3, and on performance fields (Lee & Carrasco, 2025), with an effect size of *d* = 1.41 for performance in the neutral trials. According to G*Power 3.0 (Faul, Erdfelder, Lang, & Buchner, 2007), we would need nine participants for adaptation and eight participants for performance fields to reach a power of 0.9. We also estimated the required sample size for the interaction between adaptation and location, based on a recent study between adaptation and performance fields (Lee & Carrasco, 2025) ($\eta_{p}^{2}$ = 0.34); by assuming *SD* = 1, we would need 10 subjects to reach a power of 0.9 according to the Monte Carlo simulation (1000 iterations per possible subject number). The Institutional Review Board at New York University approved the experimental procedures, and all participants provided informed consent before they started the experiment.

#### Stimuli and apparatus

The target Gabor (diameter = 4°, 5 cycles per degree [cpd], 1.25° full width at half maximum) was presented on the left, right, upper, and lower cardinal meridian locations (8° from the center to center). There were four placeholders (length = 0.16°, width = 0.06°) 0.5° away from the edge of the Gabor. The fixation cross consisted of a plus sign (length = 0.25°, width = 0.06°) at the center of the screen. The endogenous attentional cue (length = 0.75°, width = 0.2°) was presented at the center.

Participants were in a dimly lit, sound-attenuated room, with their head placed on a chinrest 57 cm away from the monitor. All stimuli were generated using MATLAB (MathWorks, Natick, MA) and the Psychophysics Toolbox (Brainard, 1997; Pelli, 1997) on a gamma-corrected 20-inch ViewSonic G220fb CRT monitor (ViewSonic Corporation, Brea, CA) with a spatial resolution of 1280 × 960 pixels and a refresh rate of 100 Hz. To ensure fixation, the eye movements of each participant were recorded using an EyeLink 1000 (SR Research, Ottawa, ON, Canada) with a sample rate of 1000 Hz.

#### Experimental design and procedures

**Figure 2.**
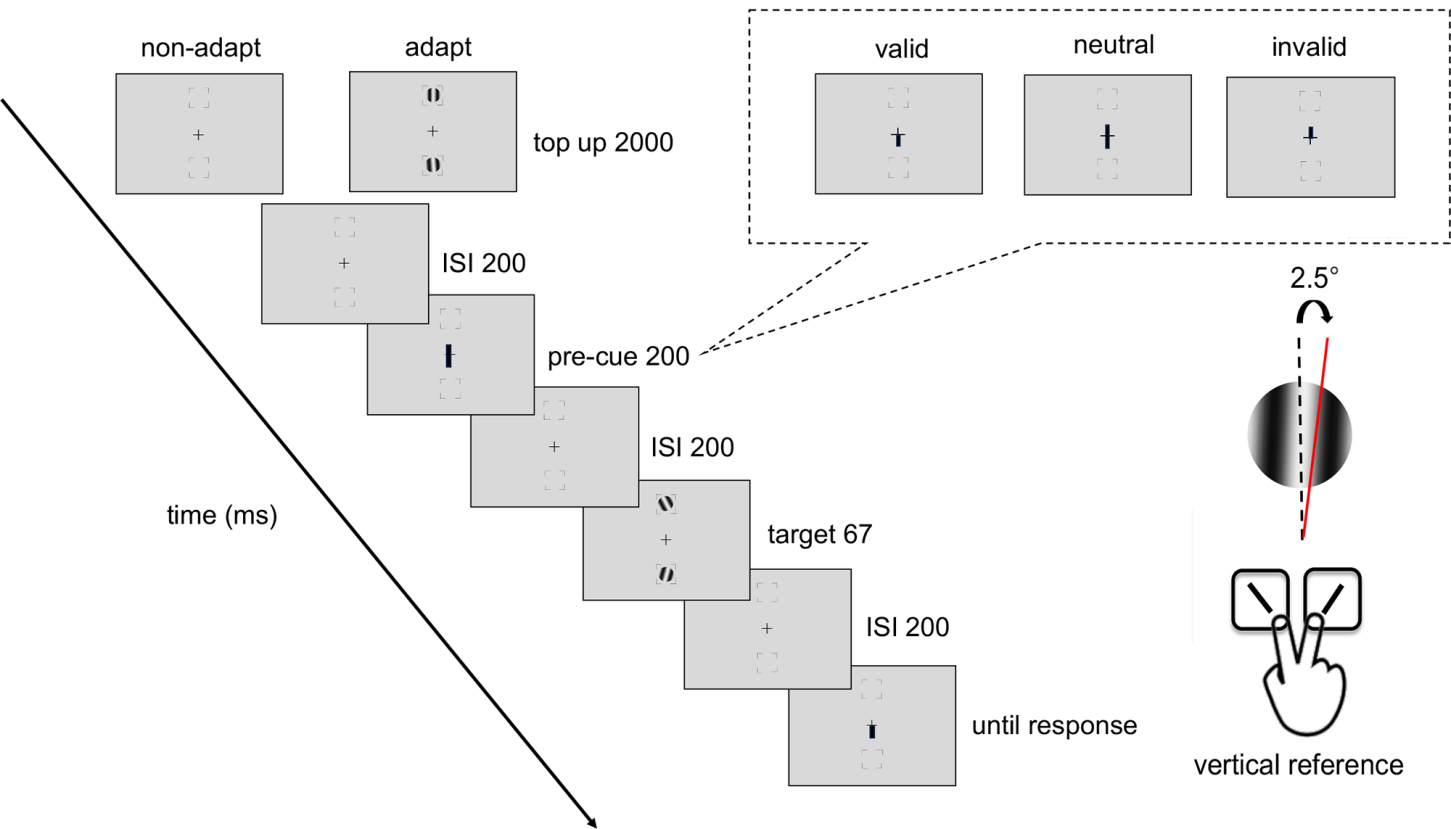
Experimental procedure: Participants performed either adaptation or nonadaptation blocks, each in separate experimental sessions. The target Gabor stimulus was always presented within the black placeholder, and target meridians were blocked. The target, two vertical Gabor stimuli, were presented 8° away from the center (e.g., at the vertical meridian here; at the horizontal meridian in a different block). Participants were instructed to respond whether the Gabor was tilted clockwise or counterclockwise from vertical. The pre-cue matched (valid condition), mismatched (invalid condition) the response cue, or did not provide location information (neutral condition). For illustration purposes, the stimulus size and spatial frequency shown here are not to scale.

Figure 2 shows the procedure of titration and the endogenous attention task. In the adapted condition, at the beginning of each block, participants adapted to a vertical 5-cpd Gabor patch. flickering at 7.5 Hz in a counterphase manner, presented at the target location for 60 seconds. Each trial started with a 2-second top-up phase to ensure a continuous adaptation effect throughout the block. In the non-adaptation condition, participants maintained fixation at the center for 4 seconds (without Gabor) at the beginning of each block and for 2 seconds at the beginning of each trial.

After the top-up, there was a 200-ms interstimulus interval (ISI) before an endogenous pre-cue was presented for 100 ms. Following a 200-ms ISI, the tilted Gabor was then presented for 67 ms, followed by another 200-ms ISI and then the response cue. In a valid trial, the location indicated by the response cue matched the precue; in an invalid trial, they mismatched. In a neutral cue condition, the pre-cue pointed at both locations. Participants had to judge whether the target Gabor was tilted clockwise or counterclockwise off vertical. The tilt angle was 2.5°, based on pilot data and our previous study (Lee & Carrasco, 2025), to ensure an adaptation effect while avoiding floor or ceiling performance.

A feedback tone was presented when participants gave an incorrect response. The target locations were blocked in a horizontal block or a vertical block, where the target locations were presented at the horizontal or vertical meridians, respectively. Participants were asked to respond as accurately as possible while fixating at the center of the screen throughout the trial. A trial would be interrupted and repeated at the end of the block if a participant’s eye position deviated ≥1.5° from the center, from the pre-cue onset until the response cue onset.

Participants completed the adapted and non-adapted attentional task on the vertical and the horizontal meridian on different days, with a counterbalanced order. The order of horizontal and vertical meridian blocks was randomized, and the adaptation and non-adaptation titrations were implemented on different days, with a counterbalanced order. There were four independent staircases for each adaptation condition and location, varying Gabor contrast from 2% to 85% to reach ∼75% accuracy for the orientation discrimination task. Each staircase started from four different points (85%, 2%, the median contrast of 43.5%, and a random point between 2% and 85%) and contained 48 trials. Four blocks (192 trials per location for each adaptation and non-adaptation conditions) were conducted consecutively for the horizontal meridian block or the vertical meridian block. The contrast threshold was derived using an adaptive staircase procedure using the Palamedes toolbox (Prins & Kingdom, 2018), as in previous studies (e.g., Fernández & Carrasco, 2020; Hanning et al., 2022; Jigo & Carrasco, 2018; Lee & Carrasco, 2025; Lee et al., 2024) and averaging the last eight trials. The Gabors were always preceded by a neutral pre-cue, which, as in many studies (e.g., Dosher & Lu, 2000a; Fernández et al., 2022; Huang, Liao, Chen, & Chen, 2025; Jigo & Carrasco, 2020; Li, Pan, & Carrasco, 2021; Luzardo & Yeshurun, 2025; Palmieri & Carrasco, 2024; Ramamurthy, White, & Yeatman, 2024; Tünçok, Carrasco, & Winawer, 2025), provided the same temporal information as the valid and invalid cues but no information about the spatial location.

In this endogenous attention task, for each adapted and non-adapted condition, 20% of the trials had a neutral cue, which pointed at both locations; 80% of the trials had an attentional cue pointing toward a location (75% valid cues and 25% invalid cues). All participants completed a practice session to familiarize themselves with the task procedure.

#### Psychometric function fitting

We fitted a Weibull function for the accuracy as a function of contrast threshold. For each location and adaptation condition, a logistic function was fit to the data using maximum likelihood estimation using the fmincon function in MATLAB. The results derived from the psychometric function estimation positively correlated (*p* < 0*.*01) with the staircase results in all experiments, verifying our procedure in all conditions.

#### Behavioral data analyses

Behavioral data analyses were performed using R (R Foundation for Statistical Computing, Vienna, Austria). A three-way repeated-measures analysis of variance (ANOVA) on *d*′ was conducted on the factors of location (horizontal meridian, upper, lower), adaptation (adapted, non-adapted), and attention (valid, neutral, invalid) conditions to assess statistical significance. Repeated-measures ANOVAs along with effect size (η^2^) were computed in R and used to assess statistical significance.

### Results

#### Adaptation effect varied around polar angle

**Figure 3.**
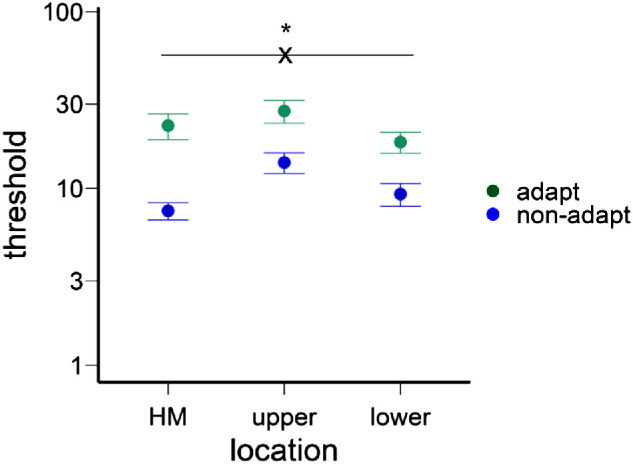
The contrast thresholds for different locations and adaptation conditions. The thresholds were higher in the vertical than horizontal meridian (HM), and higher in the upper than lower vertical meridians. The thresholds were also higher in the adapted than non-adapted conditions. Critically, the adaptation effect was stronger in the horizontal than vertical meridians. The error bars indicate ±1 *SEM*.

After deriving the C_50_ contrast for the horizontal meridian, upper vertical meridian, and lower vertical meridian for both the adapted and non-adapted conditions, we conducted a two-way ANOVA on contrast thresholds (Figure 3). This analysis showed a main effect of location, *F*(2, 22) = 7.89, *p* = 0.003, $\eta_{p}^{2}$ = 0.42, and a higher threshold in the adapted than non-adapted conditions, *F*(1, 11) = 18.44, *p* = 0.001, $\eta_{p}^{2}$ = 0.63, as well as an interaction, *F*(2, 22) = 3.58, *p* = 0.045, $\eta_{p}^{2}$ = 0.25, indicating that the adaptation effect varied across locations. We confirmed that the HVA and VMA emerged in the non-adaptation condition (Figure 3): Contrast thresholds were lower along the horizontal than the vertical meridian, *t*(11) = 5.87, *p* < 0.001, *d* = 1.69, and lower at the lower than upper vertical meridian, *t*(11) = 2.37, *p* = 0.037, *d* = 0.68.

Next, we assessed the adaptation effect at the horizontal and vertical meridians. The normalized adaptation effect (calculated as the difference between adapted and non-adapted thresholds divided by the sum of the thresholds, as in Lee & Carrasco, 2025) was stronger at the horizontal than the vertical meridian, *t*(11) = 3.39, *p* = 0.006, *d* = 0.98 (Figure 3; see gaps between adapt and non-adapt conditions for different locations), but there was no significant difference between the upper and lower vertical meridians, *t*(11) < 1.

#### Endogenous attentional effect

**Figure 4.**
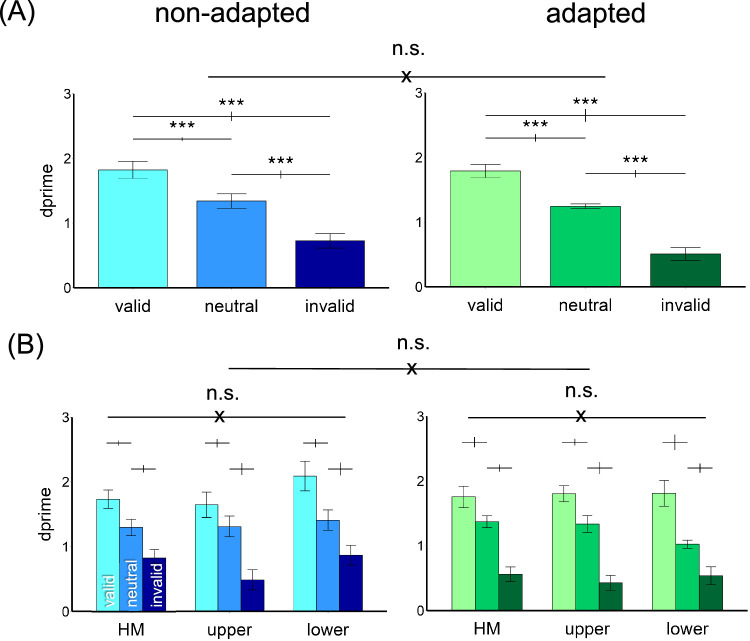
Performance in Experiment 1. (**A**) The *d*′ value was higher in the valid condition followed by the neutral and invalid conditions in both non-adapted and adapted conditions. There was no difference between the adapted and non-adapted conditions. (**B**) The attentional effects were similar around polar angle—horizontal meridian (HM) and upper and lower vertical meridians—and were comparable in the adapted and non-adapted conditions. The error bars above the bar plots indicate ±1 *SEM* of the difference between conditions. ***p < 0.001; n.s., *p* > 0.05.

Figure 4 shows the results. We compared the endogenous attentional effect on *d*′ by conducting a three-way ANOVA on the factors of location (horizontal, upper, lower meridians), attentional validity (valid, neutral, invalid), and adaptation (adaptation, non-adaptation). Given that we titrated the contrast thresholds across locations and adaptation conditions, we expected no main effects of either adaptation or location. Indeed, there was a main effect of attention, *F*(2, 22) = 53.18, *p* < 0.001, $\eta_{p}^{2}$ = 0.83, but not of location, *F*(2, 22) < 1, or of adaptation, *F*(1, 11) < 1. There was neither a three-way interaction nor two-way interaction (all *p* *>* 0.1).

**Figure 5.**
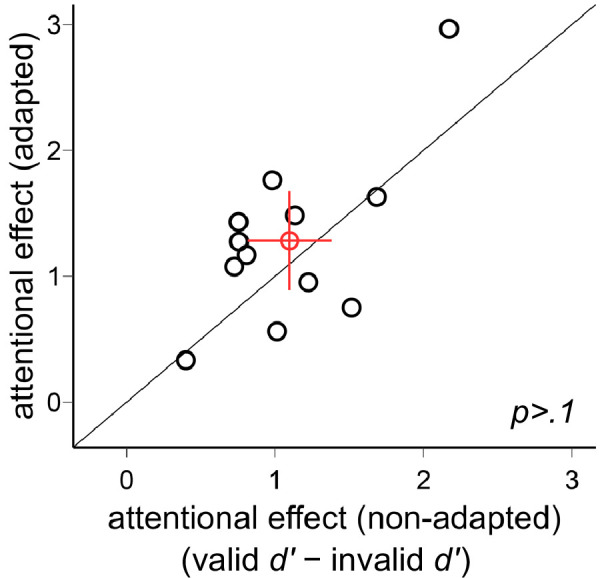
Comparison of the endogenous attentional effects (valid *d*′ − invalid *d*′) in Experiment 1. The attentional effects were comparable in the adapted and non-adapted conditions. The red circle indicates the mean of all participants, and the error bars indicate ±1 *SEM*.

The results were further confirmed by separating the adapted and non-adapted conditions into two two-way ANOVAs on attention and location. For the non-adapted condition, we observed a main effect of attention, *F*(2, 22) = 46.74, *p* < 0.001, $\eta_{p}^{2}$ = 0.81, but not of location, *F*(2, 22) < 1, or an interaction, *F*(4, 44) = 1.68, *p* > 0.1. The same pattern emerged for the adapted condition, with a main effect of attention, *F*(2, 22) = 38.59, *p* < 0.001, $\eta_{p}^{2}$ = 0.78, but not of location, *F*(2,22) < 1, or interaction, *F*(4, 44) = 1.48, *p* *>* 0.1. Thus, neither adaptation state nor location modulated the pronounced overall effect of attention. In Figure 5, we plotted the individual data for the endogenous attentional effect (valid *d′* − invalid *d′*) in the adapted and non-adapted conditions. There was no difference between the two conditions, *t*(11) = 1.27, *p* > 0.1. In sum, the endogenous attentional effect was comparable across locations and adaptation conditions.

## Experiment 2: Exogenous attention

Experiment 1 showed that endogenous attention does not reshape the performance fields, even after differential adaptation effects across meridians. In Experiment 2, we examined whether exogenous attention exhibits a similar or distinct pattern compared with endogenous attention, given their well-established differences in temporal dynamics: Whereas endogenous attention takes about 300 ms to deploy and its effects can be sustained for many seconds, exogenous attention effects peak at about 120 ms and their effects are transient (for reviews, see Carrasco, 2011; Carrasco, 2014; Carrasco & Barbot, 2014). Moreover, endogenous attention is flexible, whereas exogenous attention is not (e.g., Barbot & Carrasco, 2017; Barbot et al., 2012; Giordano et al., 2009; Hein et al., 2006; Nakayama & Mackeben, 1989; Yantis & Jonides, 1996; Yeshurun & Carrasco, 1998; Yeshurun et al., 2008), and the effects of endogenous attention scale with cue validity, whereas those of exogenous attention do not (e.g., Giordano et al., 2009; Kinchla, 1980; Mangun & Hillyard, 1990; Sperling & Melchner, 1978).

**Figure 6.**
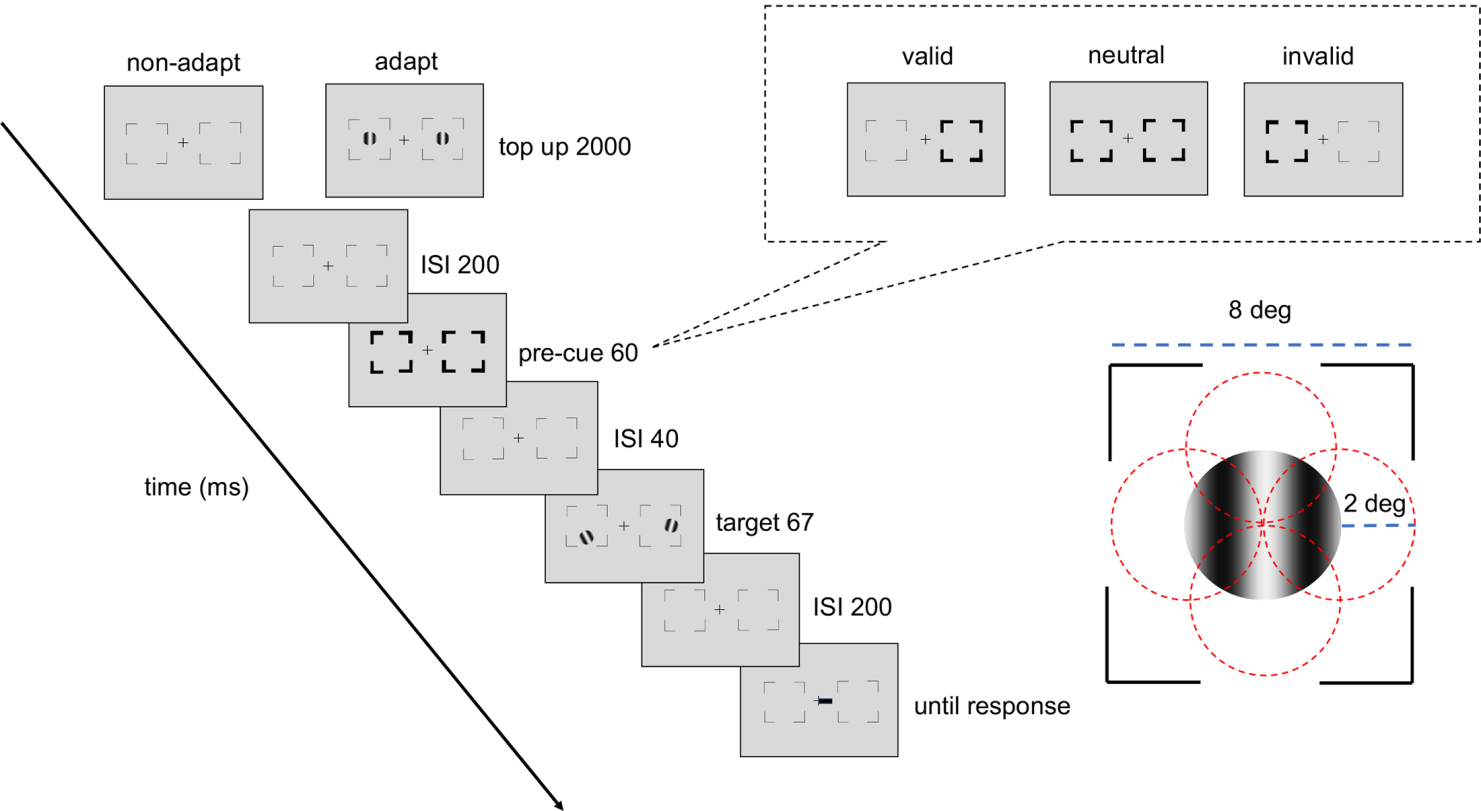
Experimental procedure. The procedure was the same as Experiment 1 except for the pre-cue and ISI timings. The pre-cue (bolded placeholders) matched (valid condition) or mismatched (invalid condition) the response cue, or it did not provide location information (neutral condition). The placeholders were wider (8°) than in Experiment 1. The target, two vertical Gabor stimuli, were presented on average 8° away from the center (e.g., at the horizontal meridian as shown here; at the vertical meridian in a different block). There were five possible target locations, which were 2° away from the central Gabor. For illustration purposes, the stimulus size and spatial frequency shown here are not to scale.

To manipulate exogenous attention, we used a peripheral cue (a bolded placeholder) presented before the target onset. According to a normalization model of attention, exogenous attention can also affect contrast gain when the attentional window is large enough (Herrmann et al., 2010; Reynolds & Heeger, 2009). To induce a large attentional window while maintaining overlap between the target and adaptors and to ensure the adaptation effect, we randomly presented the target in one of the five locations within the placeholders (Figure 6), and participants were explicitly instructed to attend to the whole space encompassed by the placeholder, as the target could appear anywhere within the placeholder. This procedure has been successfully used to manipulate the size of the attentional window in both exogenous and endogenous covert spatial attention, as well as in presaccadic attention (e.g., Binda & Murray, 2015; Cutrone, Heeger, & Carrasco, 2018; Feng & Spence, 2017; Grubb et al., 2013; Herrmann et al., 2010; Li et al., 2021).

### Methods

#### Participants

Eleven out of 12 participants^1^ who participated in Experiment 1, including author H-HL, also participated in Experiment 2. We tested the same group of participants to compare the results from endogenous and exogenous attentional effects after adaptation.

#### Stimuli and apparatus

Figure 6 shows an experimental trial. The target stimuli and the apparatus were the same as Experiment 1. The placeholders in Experiment 2 (length = 0.256° for placeholders that were farther away from the center, and length = 0.192° for placeholders that were closer to the center; all had width = 0.06°) were larger, given that there were five possible target locations: center and 2° on the upper, lower, left, or right of the central Gabor. During the cue presentation, the placeholders became thicker (6 pixels bigger for the frame elements closer to the center and 8 pixels bigger for the frame elements farther away from the center) to capture participants’ exogenous attention.

#### Experimental design and procedures

The same C_50_ contrast derived from Experiment 1 was used in Experiment 2 for the adapted and non-adapted conditions across locations. The experimental design and procedures were the same as in Experiment 1, except for the following: After the top-up, there was a 200-ms ISI before the exogenous pre-cue appeared for 60 ms, followed by a 40-ms ISI. The tilted Gabor was then presented for 67 ms followed by another 200-ms ISI and the response cue. Participants were explicitly told that the exogenous cues were not informative; that is, they were equally likely to be valid, neutral, or invalid (33% each). Participants were instructed to enlarge their attentional window during the task, as they were explicitly told that the target could appear anywhere within the placeholders.

### Results

**Figure 7.**
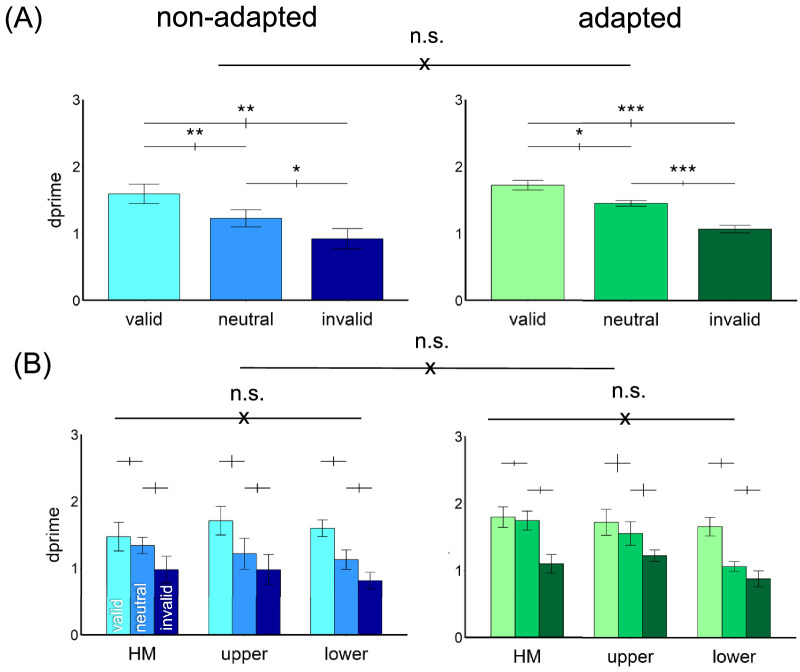
Performance in Experiment 2. (**A**) The *d*′ value was higher in the valid condition followed by the neutral and invalid conditions in both non-adapted and adapted conditions. There was no difference between the adapted and non-adapted conditions. (**B**) The attentional effects were similar around polar angle—horizontal meridian (HM), and upper and lower vertical meridians—and were comparable in the adapted and non-adapted conditions. The error bars above the bar plots indicate ±1 *SEM* of the difference between conditions. ***p < 0.001, **p < 0.01, *p < 0.05; n.s., *p* > 0.05.

Figure 7 shows our results. As in Experiment 1, we compared the exogenous attentional effect on *d*′ by conducting a three-way ANOVA on the factors of location (horizontal, upper, lower meridians), attentional validity (valid, neutral, invalid), and adaptation (adaptation, non-adaptation). There was a main effect of attention, *F*(2, 20) = 20.7, *p* < 0.001, $\eta_{p}^{2}$ = 0.67, but not of location, *F*(2, 20) = 2.61, *p* = 0.099, or adaptation, *F*(1, 10) = 1.08, *p* > 0.1. There was neither a three-way interaction, *F*(4, 40) = 2.36, *p* = 0.069, nor a two-way interaction (all *p* > 0.1).

The results here were further confirmed by separating the adapted and non-adapted conditions into two two-way ANOVAs on attention and location. For the non-adapted condition, we observed a main effect of attention, *F*(2, 20) = 13.33, *p* < 0.001, $\eta_{p}^{2}$ = 0.57, but not of location, *F*(2, 20) < 1, or interaction, *F*(4, 40) = 1.33, *p* > 0.1. The same pattern emerged for the adapted condition, with a main effect of attention, *F*(2, 20) = 21.19, *p* < 0.001, $\eta_{p}^{2}$ = 0.68, but not of location, *F*(2, 20) = 2.33, *p* > 0.1, or interaction, *F*(4, 40) = 2.01, *p* > 0.1.

**Figure 8.**
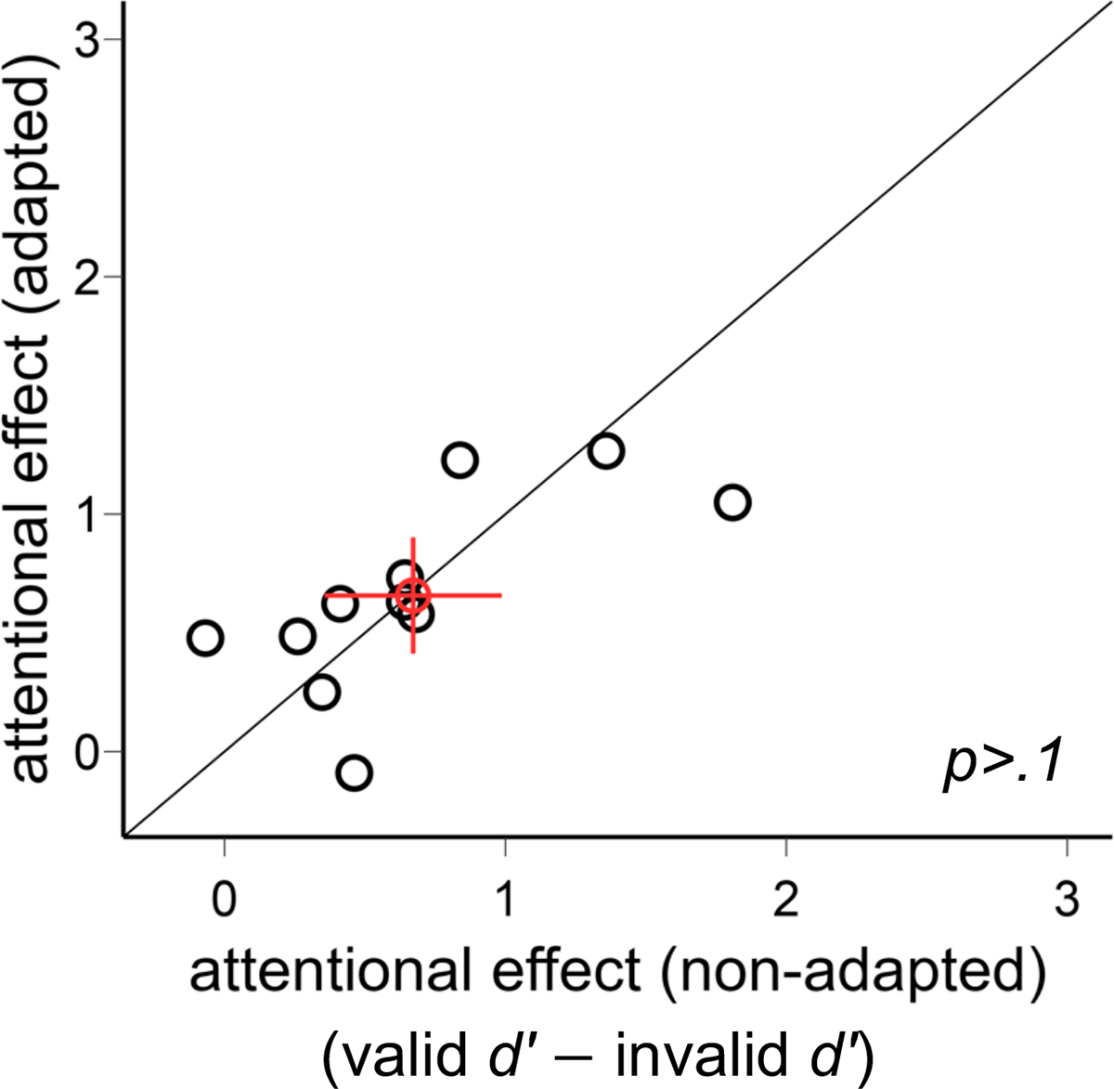
Comparison of the exogenous attentional effects (valid *d*′ − invalid *d*′) in Experiment 2. The attentional effects were comparable in the adapted and non-adapted conditions. The red circle indicates the mean of all participants, and the error bars indicate ±1 *SEM*.

The individual data for the exogenous attentional effect (valid *d*′ − invalid *d*′) in the adapted and non-adapted conditions are plotted in Figure 8. There was no difference between the two conditions, *t*(10) < 1. In sum, the exogenous attentional effect was comparable across locations and adaptation conditions.

### Comparing experiments 1 and 2

Given that we had 11 common participants in Experiments 1 and 2, we conducted a four-way repeated-measures within-subject ANOVA on the factor of type of attention (endogenous, exogenous), attentional validity (valid, neutral, invalid), adaptation (adapted, non-adapted), and location (horizontal, upper, lower meridians). There was a main effect of attentional validity, *F*(2, 20) = 51.72, *p* < 0.001, $\eta_{p}^{2}$ = 0.84, and an interaction between attentional validity and type of attention, *F*(2, 20) = 7.38, *p* = 0.004, $\eta_{p}^{2}$ = 0.42. Post hoc analyses indicated that the valid condition had the highest *d*′ followed by the neutral condition—valid – neutral, *t*(10) = 6.78, *p* < 0.001, *d* = 2.04—and invalid condition—neutral – invalid, *t*(10) = 6.17, *p* < 0.001, *d* = 1.86. The attentional effect (valid *d*′ – invalid *d*′) was stronger for endogenous than exogenous attention, *t*(10) = 2.95, *p* = 0.015, *d* = 0.89. Importantly, there was no four-way interaction, *F*(4, 40) < 1, nor were there any other significant effects (all *p* > 0.05), indicating that the effect for both types of attention did not vary across locations nor across adaptation conditions. Furthermore, we found a positive Pearson correlation (*r* = 0.39, *p* = 0.025) between the exogenous and endogenous overall attentional effect (collapsing across adaptation conditions and locations), which indicates that those observers who had a stronger effect of one type of attention also had a stronger effect for the other type.

## Discussion

In this study, we investigated whether attention interacts with adaptation around polar angle. Our results are consistent with separate studies, as they showed that, without adaptation, the typical performance fields emerged, with lower contrast thresholds at the horizontal than the vertical meridian (HVA) and at the lower than the upper vertical meridian (VMA) (e.g., Abrams et al., 2012; Baldwin et al., 2012; Cameron et al., 2002; Carrasco et al., 2001; Corbett & Carrasco, 2011; Fuller et al., 2008; Himmelberg et al., 2020; Lee & Carrasco, 2025). Also, adaptation effects were stronger at the horizontal than the vertical meridian (Lee & Carrasco, 2025), and both endogenous attention (Purokayastha et al., 2021; Tünçok et al., 2025) and exogenous attention (Cameron et al., 2002; Carrasco et al., 2001; Roberts et al., 2016; Roberts et al., 2018) enhanced contrast sensitivity similarly across all tested locations. Furthermore, our results revealed that endogenous attention restored contrast sensitivity following adaptation, and endogenous attention and exogenous attention had similar effects on contrast sensitivity before and after adaptation, as both enhanced contrast sensitivity at the attended location, with concomitant costs at unattended locations. They did so uniformly at the cardinal meridians around the visual field, despite differential adaptation effects.

The finding that endogenous attention enhanced contrast sensitivity to a similar extent in adapted and non-adapted conditions indicates that visual adaptation does not modulate the attentional effect. This novel finding is consistent with corresponding findings for exogenous attention on contrast sensitivity after adaptation (Lee et al., 2024; Pestilli et al., 2007). Despite its flexible nature (for reviews, see Carrasco, 2011; Carrasco, 2014; Carrasco & Barbot, 2014; Olivers, 2025), endogenous attention neither increased nor decreased contrast sensitivity differentially before and after adaptation, indicating that these two processes, which help manage limited bioenergetic resources, play independent roles in shaping performance.

Typically, the effect of exogenous attention manifests as response gain and the effect of endogenous attention as contrast gain (Ling & Carrasco, 2006a; Pestilli et al., 2009). In the exogenous attention experiment, we induced contrast gain by manipulating the size of the attentional window, presenting the target Gabor at one of five different locations within a larger stimulus placeholder. According to the normalization model of attention proposed by Reynolds and Heeger (2009), attention produces contrast gain rather than response gain when the attentional window is large relative to stimulus size (Reynolds & Heeger, 2009), a prediction confirmed psychophysically and with functional magnetic resonance imaging (fMRI) (Herrmann et al., 2010). By contrast, endogenous attention can induce response gain when deployed over a relatively smaller attentional window than the stimulus size (Fernández et al., 2023; Herrmann et al., 2010; Morrone, Denti, & Spinelli, 2004). Consistent with previous findings (Lee et al., 2024; Pestilli et al., 2007), exogenous attention modulated contrast sensitivity to a similar extent in adapted and non-adapted conditions, indicating that adaptation did not modulate its effect. These results support Hypothesis 1: After adaptation, covert spatial attention modulates contrast sensitivity to the same extent as without adaptation (Figure 1A).

In this exogenous attention experiment, to induce a larger attentional window, participants were explicitly told that the target could appear anywhere within the placeholders. This manipulation should not have affected the effects of exogenous attention, as it cannot induce endogenous attention. The effects of endogenous attention scale with cue validity (e.g., Giordano et al., 2009; Kinchla, 1980; Mangun & Hillyard, 1990; Sperling & Melchner, 1978), and, in the exogenous attention experiment, the cue was uninformative: Each of the valid, invalid, and neutral cues was presented on 33% of the trials, so when a cue indicated one location out of two, its validity was 50%. Thus, had observers deployed endogenous attention in Experiment 2, performance would have been similar for valid and invalid conditions. Instead, we found significant benefits at the attended location and significant costs at unattended locations, consistent with an exogenous attention effect. Moreover, given the timing of the exogenous cue (∼120 ms) and that endogenous attention requires ∼300 ms to be deployed (e.g., Cheal, Lyon, & Hubbard, 1991; Geweke, Pokta, & Störmer, 2021; Liu, Stevens, & Carrasco, 2007; Nakayama & Mackeben, 1989; Remington, Johnston, & Yantis, 1992; for reviews, see Carrasco, 2011; Carrasco, 2014; Carrasco & Barbot, 2014), endogenous attention could not contribute.

Adaptation was more pronounced at the horizontal than the vertical meridian. Unlike our previous study (Lee & Carrasco, 2025), which blocked each target location, here we introduced greater target uncertainty by using two possible target locations per trial (Figure 2). The replication of the adaptation pattern across studies shows that that the previous findings are robust to target uncertainty and generalize across participants. Most adaptation studies have examined only the horizontal meridian (e.g., Beaton & Blakemore, 1981; Carrasco et al., 2006; Gao, Webster, & Jiang, 2019; Greenlee, Georgeson, Magnussen, & Harris, 1991; Pestilli et al., 2007; Schieting & Spillmann, 1987) or only the vertical meridian (e.g., Bell, Gheorghiu, Hess, & Kingdom, 2011; Bell, Gheorghiu, & Kingdom, 2009; Montaser-Kouhsari & Rajimehr, 2004), or did not analyze target locations separately (e.g., Bao, Fast, Mesik, & Engel, 2013; Lin, Zhou, Naya, Gardner, & Sun, 2021; Ling & Carrasco, 2006b; Zimmermann, Weidner, Abdollahi, & Fink, 2016). Our results add further evidence that adaptation differs across meridians, an important finding to consider in future studies and models of vision.

Endogenous and exogenous attention enhanced contrast sensitivity similarly around polar angle, despite the differential effects of adaptation. Consistent with previous studies (Cameron et al., 2002; Carrasco et al., 2001; Roberts et al., 2016; Roberts et al., 2018), asymmetries at the cardinal locations were resistant to both endogenous and exogenous attention, indicating their resilient nature and that they cannot be easily reshaped. In contrast, consistent with a recent finding (Lee & Carrasco, 2025), visual adaptation reduced contrast sensitivity more at the horizontal than the vertical meridian, yet neither type of covert spatial attention modulated the extent of the asymmetries altering the shape of the performance fields, notwithstanding the differential adaptation effect. This similar effect is notable given that endogenous attention is flexible and exogenous attention automatic (e.g., Carrasco, 2011; Carrasco, 2014; Carrasco & Barbot, 2014; Olivers, 2025), yet neither compensated for poor performance. These findings provide further evidence regarding the resilience of polar angle asymmetries and support hypothesis 4 (Figure 1D): Visual adaptation does not modulate the effects of covert spatial attention, even at the location of poorest performance.

What contributes to performance asymmetries in the HVA and VMA? These asymmetries arise from both retinal and cortical factors. Retinally, cone density is higher at the horizontal than the vertical meridian (Curcio, Sloan, Kalina, & Hendrickson, 1990; Curcio, Sloan, Packer, Hendrickson, & Kalina, 1987), and midget retinal ganglion cell (RGC) density is higher at the lower than the upper vertical meridian (Curcio et al., 1990; Song, Chui, Zhong, Elsner, & Burns, 2011). Cortically, V1 surface area is larger for the horizontal than the vertical meridian, and for the lower than the upper vertical meridian (Benson, Kupers, Barbot, Carrasco, & Winawer, 2021; Himmelberg et al., 2021; Himmelberg, Kwak, Carrasco, & Winawer, 2025; Himmelberg et al., 2023; Himmelberg, Winawer, & Carrasco, 2022; Himmelberg, Winawer, & Carrasco, 2023; Lee & Carrasco, 2025; Silva et al., 2018). Moreover, cortical factors account for more variance in these asymmetries than retinal factors (Kupers, Benson, Carrasco, & Winawer, 2022). Still, these factors cannot fully explain behavioral differences observed in psychophysical tasks, which are diminished but still present when stimulus size is cortically magnified (Jigo, Tavdy, Himmelberg, & Carrasco, 2023), suggesting that additional factors—such as sensory tuning and neuronal computations—also contribute to the HVA and VMA (Himmelberg, Winawer, et al., 2023; Jigo et al., 2023; Xue, Barbot, Abrams, Chen, & Carrasco, 2025).

Endogenous and exogenous attention rely on different neural substrates. fMRI studies show differential activity modulation across the frontoparietal network (Beck & Kastner, 2014; Buschman & Miller, 2009; Chica, Bartolomeo, & Lupiáñez, 2013; Fiebelkorn & Kastner, 2020; Kastner & Buschman, 2017; Meyer, Du, Parks, & Hopfinger, 2018), temporoparietal junction (Dugué, Merriam, Heeger, & Carrasco, 2018), and visual cortex (Dugué, Merriam, Heeger, & Carrasco, 2020; Hopfinger & West, 2006; Ling, Jehee, & Pestilli, 2015). Transcranial magnetic stimulation (TMS) studies, which disrupt the neuronal balance between excitation and inhibition (Bradley, Nydam, Dux, & Mattingley, 2022; Kobayashi & Pascual-Leone, 2003; Valero-Cabré, Pascual-Leone, & Coubard, 2011), revealed that early visual cortex plays a critical role for adaptation (Lee et al., 2024; Perini et al., 2012) and exogenous attention (Fernández & Carrasco, 2020; Lee et al., 2024), whereas the human homolog of the right frontal eye fields (rFEF+) plays a critical role for endogenous attention (Fernández et al., 2023). Critically, disrupting rFEF+ does not affect exogenous attention (Chen et al., 2025), and disrupting early visual cortex does not affect endogenous attention (Fernández et al., 2023), indicating a double dissociation. Despite these distinct neural underpinnings, both types of covert spatial attention affected contrast sensitivity uniformly at the cardinal meridians around polar angle and did not interact with location or adaptation. These findings suggest that distinct neuronal populations underlie polar angle asymmetries, adaptation, and attentional modulation.

We found stronger attentional effects for endogenous than exogenous attention. Given that adaptation is more effective when the adaptor and the target spatially overlap (Kovács, Zimmer, Harza, & Vidnyánszky, 2007; Larsson & Harrison, 2015; Webster, 2011; Webster, 2015), we introduced target uncertainty with five possible target locations and allowed 2° overlap between the adaptor and target to elicit adaptation while allowing exogenous attention to operate via contrast gain. This manipulation may have yielded a slightly narrower exogenous attentional window than for endogenous attention in our design, as well as compared with previous studies. For example, Herrmann et al. (2010) used five possible target locations with no overlap, whereas in our current study, the target Gabors could overlap by 2° within placeholders. According to the normalization model of attention proposed by Reynolds and Heeger (2009), attention multiplies stimulus-evoked activity before divisive normalization. In our task, normalization may have pooled a broader suppressive drive than in typical exogenous attention tasks, but not as broad as in typical endogenous attention tasks—leading to less pronounced contrast gain and thus weaker exogenous than endogenous attention effects.

Why do the type of spatial covert attention, adaptation, and polar angle asymmetries not interact? The visual cortex plays a crucial role in all three processes. fMRI studies have shown that covert endogenous spatial attention modulates activity in visual cortex via feedback from frontoparietal cortex (Buschman & Miller, 2009; Chica et al., 2013; Corbetta, Patel, & Shulman, 2008; Corbetta & Shulman, 2002; Dugué et al., 2020; Lauritzen, D'Esposito, Heeger, & Silver, 2009; Pestilli, Carrasco, Heeger, & Gardner, 2011) and increasingly modulates activity in the occipital visual areas (Dugué et al., 2020), with V1/V2, its early visual areas, being not critical for endogenous attention, as TMS on these areas does not alter its effect on visual perception (Fernández et al., 2023). In contrast, exogenous attention modulates visual cortex via feedforward activation (Dassanayake, Michie, & Fulham, 2016; Dugué et al., 2020; Hopfinger, Luck, & Hillyard, 2004; Liu, Pestilli, & Carrasco, 2005; Wang, Chen, Yan, Zhaoping, & Li, 2015; Westerberg, Schall, Woodman, & Maier, 2023), and V1 and V2 are critical for its effect, as TMS on these areas eliminates the effect of exogenous attention on visual perception (Fernández & Carrasco, 2020; Lee et al., 2024). Moreover, these two attention types also differentially modulate visual subregions of the temporoparietal-junction (Dugué et al., 2018). All of these differences underscore the distinct contributions of endogenous and exogenous in modulating visual perception.

Early visual cortex also plays a critical role in visual adaptation. TMS over V1/V2 decreases contrast adaptation (Perini et al., 2012), and adaptation modulates contrast response functions in V1/V2 (Altan et al., 2025; Gardner et al., 2005; Vinke, Bloem, & Ling, 2022). A TMS study revealed that adaptation and exogenous attention interact in early visual cortex (Lee et al., 2024), but it is unknown whether they do so systematically around polar angle, as several factors shape asymmetries. Moreover, it is currently unknown whether endogenous attention and adaptation interact either in early occipital or in frontal areas.

Both adaptation (Lee & Carrasco, 2025) and polar angle asymmetries (Benson et al., 2021; Himmelberg et al., 2022; Himmelberg, Winawer, et al., 2023; Lee & Carrasco, 2025) correlate with V1 surface area, but surface area alone cannot fully account for these asymmetries (Jigo et al., 2023). Additional factors such as neural gain also contribute to these asymmetries (Xue et al., 2025). Future research integrating computational modeling, neuroimaging, neurostimulation, and psychophysics will be essential to assess the relative contributions of cortical and computational factors to attention, adaptation and polar angle asymmetries.

## Conclusions

This study revealed that performance asymmetries are resistant to the effects of both endogenous and exogenous covert spatial attention, despite their distinct temporal dynamics and differences in flexibility—even after adaptation induces differential effects across meridians. Although both adaptation and attention help allocate limited resources according to task demands, neither type of covert spatial attention differentially enhances target processing at locations that differ in intrinsic discriminability and their corresponding representation in cortical surface area.

## Footnotes

1 One participant was no longer around NYC to participate.
